# Editorial: The Science and Practice of Captive Animal Welfare

**DOI:** 10.3389/fpsyg.2020.01851

**Published:** 2020-08-14

**Authors:** Bonnie M. Perdue, Sally L. Sherwen, Terry L. Maple

**Affiliations:** ^1^Department of Psychology, Agnes Scott College, Decatur, GA, United States; ^2^Zoos Vic, Melbourne, VIC, Australia; ^3^Jacksonville Zoo and Gardens, Jacksonville, FL, United States

**Keywords:** welfare, animal, captivity, zoo, enrichment

Animal welfare science is not a new field of study, but has gained immense traction in the past decades (Wilson, 1982; Novak and Suomi, 1988; Maple and Perdue, 2013; Sherwen et al., 2018). The study of animal welfare and wellness from a scientific perspective yields valuable improvements to captive care given the reliance on empirical data rather than anecdote or opinion. This shift to scientifically approaching welfare has been applied to a range of captive animal settings including farms, zoos, laboratories, and personal ownership. The extent to which the principles of animal welfare science are implemented in these settings ranges from strict, highly regulated guidelines such as those followed in research laboratories to the less or unregulated pet ownership or emotional support animals. Nonetheless, there is great potential for synergy across these settings if the data on captive animal welfare is collected in an empirical manner. Here we gather a number of studies spanning different zoo and aquarium settings with a range of species that builds our knowledge of how to adequately assess and ultimately advance animal welfare in captivity.

## Exhibit Design and Enrichment

The physical space or enclosure in which an animal lives is a universally important aspect of animal welfare (Hediger, 1950; Hancocks, 2001). This includes aspects of the structural design itself as well as components of enrichment that may be permanent or periodically included or removed. Browning and Maple offer novel perspectives on how to measure the actual space available in an animal enclosure beyond the standard measures of area. Spatial volume is rarely calculated when describing useable space, despite the fact that so many species are largely arboreal in their locomotor habits. Browning and Maple provide a methodology to measure complex space, acknowledging that there more sophisticated measurements are available to designers. Beyond the physical space, various forms of enrichment that are either permanently or temporarily added to environment have been found to have a substantial influence on animal welfare (Bacon, 2018). Fernandez and Timberlake offer an overview of how to select enrichment and evaluate its effectiveness in lemurs and find that conducting a preference assessment may be a fairly simple method for identifying food items to be used with enrichment devices. Moving beyond standard forms of enrichment, Regaiolli et al. draw from cognitive research and assess the effectiveness of visual illusions as a form of enrichment for lions. Clark et al. present a technique for providing cognitive enrichment while preserving the naturalistic design of the zoo experience and providing a “screen-free” enrichment experience for gorillas. These findings highlight the range of potential ways to measure and improve upon the physical space and enrichment offerings to captive animals.

## Human-Animal Interactions

In addition to the physical component of an animal's experience, it is well-established that social interactions have an important influence on welfare and wellness. Much of the research in this area has focused on social conspecifics, but human interactions can have potentially positive or negative effects on animal welfare and should be evaluated thoroughly (Sherwen and Hemsworth, 2019). An animal might experience contact with a caretaker, researcher, farmer, pet owner, or zoo visitor. These interactions should be carefully evaluated to minimize potential stress and maximize the potential value of these relationships. Clegg et al. investigate how a dolphin's willingness to participate in a form of interaction, specifically positive reinforcement training, might be related to the individual's overall health. Many animals have evolved to hide symptoms of illness, but willingness to participate in interaction may provide a useful metric for identifying at risk animals before other symptoms emerge.

Conversely, interactions with animals can have significant effects on visitor perceptions and attitudes toward animals. Godinez and Fernandez review the literature on how experiences at the zoo can influence perception, behavior, and conservation opportunities both on-site and post-visit. They also highlight the importance of having a true control group of non-zoo visitors in future assessments of this kind. Chiew et al. manipulated aspects of visitor experience, including proximity to animals on exhibit and extent of engagement, to assess the influence on visitor attitudes. Notably, the penguin behavior itself was related to several aspects of visitor attitudes whereas the treatments themselves were less influential in influencing the measured attitudes.

## Variety of Species Studied

Historically, a fairly limited range of species have contributed the most to our knowledge of captive animal welfare. For a variety of historical and practical reasons, much of the early research in this domain focused on farm animals such as cows and chickens, lab animals such as rats and mice, as well as non-human primates such as monkeys (Hill and Broom, 2009). More recent animal welfare science has drastically expanded upon the range of species studied. This diversity will yield better insights into the nature and study of animal welfare as well as the development of practical animal welfare guidelines for many different species.

For example, Hill and Nollens summarize the existing research on beluga whale welfare and highlight the importance and value of relationships between universities and zoological facilities. Allard et al. apply principles of welfare assessment and investigate the link between personality assessment in Blanding's Turtles and outcomes of reintroduction efforts. This work illustrates an important effort to bridge the gap between captive animal welfare and conservation. Overall, these articles, together with others in the special edition focusing on lemurs, lions, dolphins, penguins, highlight the potential for great diversity in the questions asked about animal welfare and the wide range of species that have the potential to contribute to this field as well as benefit from the findings.

## Conclusions

Animal welfare science is a rapidly growing and critically important field in our society. As illustrated in this special edition, a wide variety of approaches to measuring, improving upon, and implementing welfare exist. The most critical pathway forward is to rely on empirical evidence and strong experimental design. By doing so, we can improve our knowledge and understanding of animal welfare and optimize the lives of the animals in our care.

## Author Contributions

BP, SS, and TM shared responsibility of the editorial process and wrote, reviewed, and edited the editorial summary. All authors contributed to the article and approved the submitted version.

## Conflict of Interest

The authors declare that the research was conducted in the absence of any commercial or financial relationships that could be construed as a potential conflict of interest.
